# Accumulation of amyloid-like Aβ_1–42_ in AEL (autophagy–endosomal–lysosomal) vesicles: potential implications for plaque biogenesis

**DOI:** 10.1042/AN20130044

**Published:** 2014-03-12

**Authors:** Daijun Ling, Martha Magallanes, Paul M. Salvaterra

**Affiliations:** *Department of Neuroscience, Beckman Research Institute of City of Hope, Duarte, CA 91010, U.S.A.; †Irell and Manella Graduate School of Biological Sciences, City of Hope, Duarte, CA 91010, U.S.A.

**Keywords:** amyloid β, Alzheimer's disease, AEL (autophagy–endosomal–lysosomal) vesicle, Aβ, amyloid β, AD, Alzheimer's disease, AEL, autophagy–endosomal–lysosomal, APP, amyloid precursor protein, CathD, cathepsin D, FA, formic acid, GVD, granulovacuolar degeneration, HRP, horseradish peroxidase, RT–qPCR, reverse transcription–quantitative PCR

## Abstract

Abnormal accumulation of Aβ (amyloid β) within AEL (autophagy–endosomal–lysosomal) vesicles is a prominent neuropathological feature of AD (Alzheimer's disease), but the mechanism of accumulation within vesicles is not clear. We express secretory forms of human Aβ_1–40_ or Aβ_1–42_ in *Drosophila* neurons and observe preferential localization of Aβ_1–42_ within AEL vesicles. In young animals, Aβ_1–42_ appears to associate with plasma membrane, whereas Aβ_1–40_ does not, suggesting that recycling endocytosis may underlie its routing to AEL vesicles. Aβ_1–40_, in contrast, appears to partially localize in extracellular spaces in whole brain and is preferentially secreted by cultured neurons. As animals become older, AEL vesicles become dysfunctional, enlarge and their turnover appears delayed. Genetic inhibition of AEL function results in decreased Aβ_1–42_ accumulation. In samples from older animals, Aβ_1–42_ is broadly distributed within neurons, but only the Aβ_1–42_ within dysfunctional AEL vesicles appears to be in an amyloid-like state. Moreover, the Aβ_1–42_-containing AEL vesicles share properties with AD-like extracellular plaques. They appear to be able to relocate to extracellular spaces either as a consequence of age-dependent neurodegeneration or a non-neurodegenerative separation from host neurons by plasma membrane infolding. We propose that dysfunctional AEL vesicles may thus be the source of amyloid-like plaque accumulation in Aβ_1–42_-expressing *Drosophila* with potential relevance for AD.

## Abstract 2

Aβ_1–42_-containing plaques are an invariant phenotype of Alzheimer's disease. Using a *Drosophila* model we show selective amyloid-like Aβ_1–42_ accumulation in dysfunctional autophagy–endosomal–lysosomal vesicles. Our results suggest they may be a source of plaque in *Drosophila* with potential relevance to AD.

## INTRODUCTION

The neuropathology of AD (Alzheimer's disease) is characterized by the accumulation of extracellular amyloid plaques and intracellular neurofibrillary tangles (Perl, 2010). The plaques are composed primarily of Aβ_1–42_ peptide, whereas tangles are composed of hyperphosphorylated tau. The underlying cellular pathogenic mechanisms resulting in these cardinal hallmarks of the disease are not completely understood and even less is known about other types of AD-related neuropathology such as the early intracellular accumulation of GVD (granulovacuolar degeneration) bodies, a feature not only of AD but also other neurodegenerative diseases (Okamoto et al., 1991). GVD bodies exhibit properties of autophagic, endocytic, and lysosomal vesicles (Funk et al., 2011; Ling and Salvaterra, 2011a).

Neurons maintain especially active bidirectional membrane trafficking to and from plasma membrane via recycling endocytosis (Maxfield and McGraw, 2004). Endosomal transport vesicles mediate this process, and some ultimately converge by fusion with autophagy and lysosomal vesicles to form AEL (autophagy–endosomal-lysosomal) vesicles (Liou et al., 1997; Saftig and Klumperman, 2009; Manjithaya and Subramani, 2011). Normal AEL vesicle trafficking is essential for intraneuronal signaling, cargo degradation, protein and lipid sorting, axonal transport and synaptic plasticity (Maxfield and McGraw, 2004; Saftig and Klumperman, 2009; Manjithaya and Subramani, 2011) and their abnormal accumulation is one of the earliest events observed in AD pathology (Nixon, 2005; Nixon et al., 2005). Several AEL relevant genes such as *BIN1*, *CD2AP*, *PICALM* and *CD33* are reported to be associated with AD (Hu et al., 2011a, 2011b; Naj et al., 2011). In addition, AEL vesicles have been documented as the main reservoir of intracellular Aβ (amyloid β) peptides, thought to be causative agents of AD pathogenesis (Cataldo et al., 2004; Yu et al., 2005). These Aβ peptides are produced from a sequential proteolysis of APP (amyloid precursor protein) mediated by β- and γ-secretases resulting in Aβ peptides of 39–43 amino acids (the most common AD-associated forms are Aβ_1–40_ and Aβ_1–42_.) Since APP, PS1 (a component of the γ-secretase complex) and BACE1 (the β-secretase) have been localized, at least in part, to AEL vesicles, the vesicles may be a site of amyloidogenic APP processing (Cataldo et al., 2004; Yu et al., 2005). However, this view has been challenged by conflicting observations (Boland et al., 2010) and other studies suggest amyloidogenic APP processing occurs primarily at the plasma membrane (Armstrong, 1998; Takahashi et al., 2002b; Marchesi, 2005). The exact cellular location of Aβ production from APP proteolysis and hence amyloidogenesis is thus still not completely resolved.

In this study, we examine the involvement of the AEL pathway in Aβ_1–42_ accumulation using a well-studied *Drosophila* model of AD (Ling et al., 2009). Direct Aβ_1–40_ or Aβ_1–42_ peptide transgene expression eliminates the need for any APP proteolytic processing, an admittedly different process from that which occurs in AD, but has the advantage of allowing direct observations of peptide routing unconfounded by uncertainties in the various sites proposed for APP proteolysis. The Aβ transgenes we use both contain a secretory signal peptide that directs their initial biosynthesis to the normal cellular secretory pathway, a biosynthetic route shared with APP (Gouras et al., 2005; Laferla et al., 2007). In young flies, Aβ_1–42_ preferentially associates with cellular membranes, especially plasma membranes. Endosomal trafficking may thus account for its specific intracellular accumulation within AEL vesicles relative to Aβ_1–40_. Antibody staining with different aggregation state-specific anti-Aβ antibodies indicates that only Aβ_1–42_ within dysfunctional AEL vesicles in older animals is in an amyloid-like state. The processes of neurodegeneration or non-neurodegenerative plasma membrane infolding may relocalize dysfunctional Aβ_1–42_-containing AEL vesicles to extracellular spaces in older flies, suggesting that this could be the mechanism of plaque formation in *Drosophila*. We present a model for this possibility and discuss its potential relevance for plaque formation in AD.

## MATERIALS AND METHODS

### Fly strains and Aβ expression

*Drosophila melanogaster* were raised using standard methods. Human Aβ_1–40_ or Aβ_1–42_ was expressed in *Drosophila* central nervous system using the binary Gal4-UAS technique as illustrated in Supplementary Figures S1A and S1B (at http://www.asnneuro.org/an/006/an006e139add.htm). Fly strains used in this work (including 3.1 kb Gad1-Gal4, 7.4 kb Cha-Gal4, Elav-GeneSwitch-Gal4, UAS-Aβ_1–40_, UAS-Aβ_1–42_, UAS-GFP^S65T^, UAS-GFP-Atg8a, UAS-Atg5^RNAi^, UAS-Atg12^RNAi^, Atg1^∆3D^) were previously described (Ling et al., 2009; Ling and Salvaterra, 2011b). Additional fly strains are UAS-mCherry-Atg8a (Chang and Neufeld, 2009) and UAS-mRFP-Rab4 (obtained from the Bloomington *Drosophila* Stock Center).

### Neuron culture and measurement of Aβ secretion

Embryos at late gastrula stage were collected from Elav-GS-Gal4:UAS-GFP flies that incorporate either the UAS-Aβ_1–40_ or UAS-Aβ_1–42_ transgene. Embryonic cells were dissociated and cultured as described (Salvaterra et al., 1998). Neurons were differentiated for 5 days at 25°C in Schneider's *Drosophila* medium (Invitrogen) supplemented with 10% FBS, 0.3 μg/ml insulin, 100 U/ml penicillin and 100 μg/ml streptomycin. Culture medium was then replaced by medium with reduced serum (1%) containing 0.1 μM RU486 (Sigma) to induce Aβ expression for 5 days. Parallel cultures with no RU486 inducer served as a negative control. Culture medium was collected for measurement of extracellular Aβ secretion. Cells were harvested and lysed in ice-cold RIPA/SDS buffer [50 mM Tris/HCl (pH 8.0), 0.5% sodium deoxycholate, 1% Triton X-100, 150 mM NaCl and 1% SDS] plus protease inhibitors (Roche). Aβ in culture medium or cell lysate was immunoprecipitated using the anti-Aβ antibody 6E10 (Covance) and Protein A/G plus agarose (Santa Cruz Biotechnology). The immunoprecipitated proteins were eluted and separated by electrophoresis through a 4–20% Tris-glycine gel (Invitrogen), transferred to PVDF membranes and detected using anti-Aβ 6E10 as the primary antibody and HRP (horseradish peroxidase)-conjugated chicken anti-mouse IgG (Santa Cruz Biotechnology) secondary antibody following incubation with SuperSignal West Pico chemiluminescent substrate (Pierce). Densitometric analysis of Western blot bands was performed using ImageJ (NIH). The densitometry of Aβ bands were normalized by the immunoprecipitation antibody bands at 50 kDa (Figures 2C and 2E). For correction of the Aβ species-specific sensitivity to the primary antibody, 1 pmol of synthetic Aβ_1–40_ or Aβ_1–42_ (California Peptide Research) was added to the negative control samples as an Aβ species-specific calibrator.

### Cathepsin D activity assay

Frozen heads from 30 adult females were homogenized in 100 μl of ice-cold buffer [10 mM Tris (pH 7.5), 5 mM EDTA and 0.25 M sucrose] and centrifuged at 20000 ***g*** for 10 min at 4°C to remove debris, nuclei and large mitochondria. The supernatant was centrifuged at 200000 ***g*** for 45 min to collect microsomal membranes [i.e. lysosomes, autolysosomes, plasma membranes, ER (endoplasmic reticulum), Golgi, etc.]. The pellet was dissolved in 100 μl of 0.1M sodium acetate buffer (pH 5.0) containing 0.1% Triton X-100. CathD (cathepsin D) activity was measured as described (Yasuda et al., 1999) using a fluorescently labeled CathD substrate. Samples were incubated at 35°C in sodium acetate (pH 3.5) for up to 3 h and fluorescence was measured using a SpectraMax microplate reader at 328 nm (excitation) and 393 nm (emission). Enzyme activity was averaged from multiple independently prepared replicates and presented as arbitrary relative fluorescence units/h per μg of protein. Enzyme assays were linear with respect to sample and incubation time. Total proteins were measured using a BCA assay (Pierce) for data normalization.

### Western blotting assay

Fly heads from 25-day-old females were homogenized in RIPA/SDS buffer [50 mM Tris/HCl (pH 8.0), 0.5% sodium deoxycholate, 1% Triton X-100, 150 mM NaCl and 1% SDS] with protease inhibitors (Roche Complete). Samples were incubated for 1 h at 4°C and centrifuged at 12000 ***g*** for 20 min. The supernatant contained RIPA/SDS-soluble Aβ_1–42_. Pellets were washed with RIPA buffer, homogenized in 70% FA (formic acid), incubated 1 h at 4°C and centrifuged at 12000 ***g*** for 20 min. The supernatant was collected, dried using a SpeedVac centrifuge (Savant) and dissolved in DMSO to obtain FA-soluble Aβ_1–42_. Proteins were separated on 4–20% polyacrylamide gradient gels (Invitrogen), transferred on to PVDF membranes (Bio-Rad Laboratories), detected by anti-Aβ 6E10 antibody staining (Covance) and HRP-conjugated secondary antibody, and visualized with SuperSignal West Pico chemiluminescent substrate (Pierce) to expose X-ray film. Densitometry of Aβ or loading control bands (40 kDa unspecific protein) was performed using Bio-Rad Quantity-One software.

### Immunohistochemistry

Dissected brains were fixed in 4% paraformaldehyde at 4°C overnight. For immunostaining using anti-Aβ antibody 4G8 (Covance) or rabbit polyclonal anti-Aβ_1–42_ antibody ab12267 (AbCam), brains were pre-treated with 70% FA for 10 min. For immunostaining using aggregated-Aβ-specific antibody 7A1a (New England Rare Reagents), FA pre-treatment was not included. Primary antibody immunostaining was detected using Alexa Fluor-555-conjugated secondary antibody (Invitrogen). For immunostaining with anti-Rab5 antibody (AbCam), the secondary antibody used for detection was Alexa Fluor-647-conjugated goat anti-rabbit IgG (Invitrogen). Samples were observed and images collected using confocal microscopy (Zeiss LSM 510).

### Congo Red histochemical staining

Fly heads were fixed in 4% paraformaldehyde at 4°C for 24 h then washed thoroughly in PBS followed by infiltration with graded concentrations of sucrose. Cryosections were stained with Congo Red (Sigma) following a published procedure (Wilcock et al., 2006). Microscopy images were collected using a 60× objective for both bright-field and dark-field illumination under polarized light.

### Fluorescent Congo Red, LysoTracker and CellMask imaging

For Congo Red fluorescence imaging, the brains were fixed and washed as above then stained with 0.2 mg/ml Congo Red in PBS for 10 min. The Congo Red solution was freshly prepared and filtered through a 0.2 μm filter before use. After PBS washing, brains were observed using confocal microscopy. Congo Red fluorescence was detected as described (Wiesehan et al., 2003). LysoTracker red (Molecular Probes) staining was performed as previously described (Ling et al., 2009). For the CellMask plasma membrane staining, whole brains were first immunostained using anti-Aβ 4G8 (Covance) and Alexa Fluor-555-conjugated goat anti-mouse IgG (Invitrogen) secondary antibody, then washed in PBS, followed by a 10 min incubation in PBS containing 5 μg/ml CellMask™ Deep Red plasma membrane stain (Invitrogen). The brains were not permeabilized with detergent prior to the CellMask staining. After 5×PBS washing, brains were mounted and observed by confocal microscopy.

### Fluorescence and electron microscopy

Expression of UAS-fluorescent protein transgenes (cytosolic GFP, mCherry-Atg8a, GFP-Atg8a, and mRFP-Rab4) was controlled by a Gal4 driver and fluorescence detected using confocal microscopy on adult brains fixed in PBS with 4% formaldehyde for 30 min, followed by thorough washing in PBS. Electron microscopy was performed as previously described (Ling et al., 2009) using an FEI Tecnai transmission electron microscope. Independent observations from three to five animals were performed for each experimental condition.

### Co-localization analysis

Transgenic GFP-Atg8a and mRFP-Rab4 were expressed in neurons of control, Aβ_1–40_ and Aβ_1–42_ flies. The brains from age-matched female adults were dissected, fixed and observed using confocal microscopy. Kenyon cells from *Drosophila* mushroom bodies were selected as the area of interest due to high neuronal density in this brain region. Z-stack images with multiple channels (green for GFP-Atg8a and red for mRFP-Rab4) were collected using a 63× (NA=1.2) water-immersion objective. At least ten optical sections with z-spacing of 0.5 μm were acquired. Image collection from all samples used identical microscopy conditions including the size of the pinhole, optimized contrast and detector gain. Two independent z-stacks were collected from each brain sample and at least three independent brains were observed for each experimental condition. Co-localization assays were performed using ImageJ (NIH) combined with the Intensity Correlation Analysis plugin (Li et al., 2004). Mander's overlap coefficient was used to quantify co-localization of GFP-Atg8a and mRFP-Rab4 puncta. Mander's overlap coefficient ranges from 0 (no co-localization) to 1 (100% co-localization).

### RT–qPCR (reverse transcription–quantitative PCR)

RT-qPCR was performed as described (Ling and Salvaterra, 2011c). The primers for Aβ_1–40_ and Aβ_1–42_ transgenes were 5′-ATGAGTCCAATGATTGCACCT-3′ and 5′-AGACTTTGCATCTGGCTGCTA-3′. Ten reference genes [*14-3-3ε*, *Appl*, *Cyp1*, *Elav*, *eIF-1A*, *l (3)02640*, *Rap2l*, *RpL32*, *Su (Tpl)* and *αTub84B*] were measured for data-specific normalization (Ling and Salvaterra, 2011c). *C*_T_ values from real-time PCR were analyzed using a custom SASqPCR program (Ling, 2012). Mean normalized expression ratios were calculated as described (Ling et al., 2012).

### Statistical analysis

**Figure 1:**
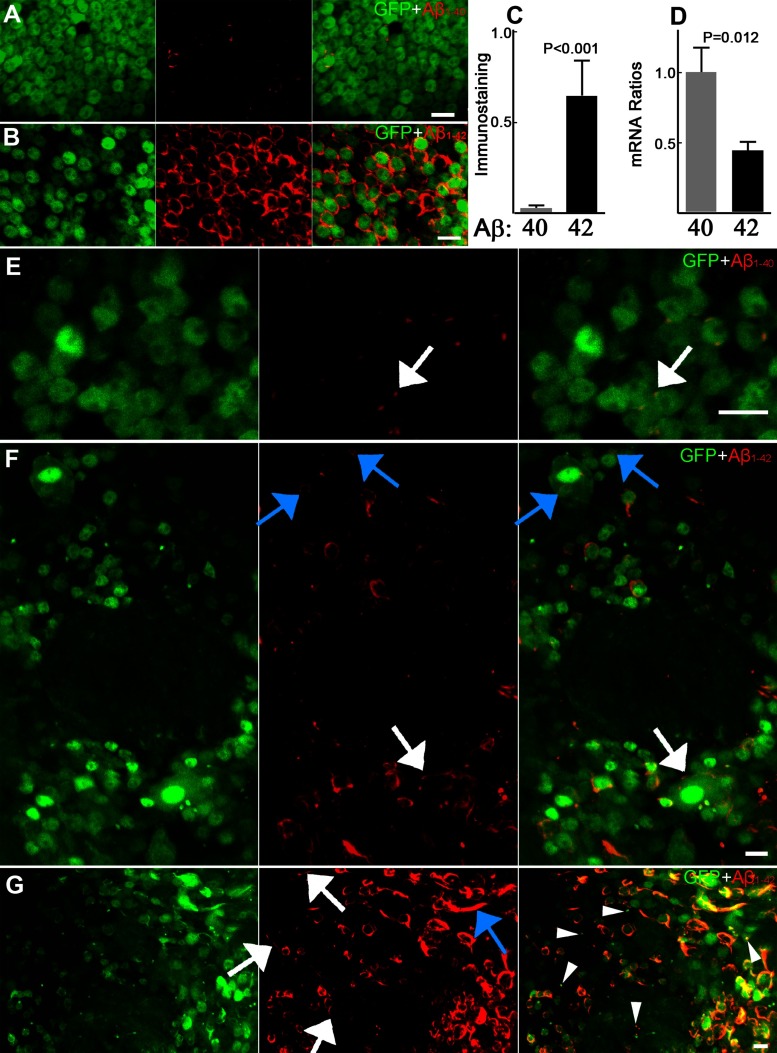
Differential accumulation of Aβ_1–40_ and Aβ_1–42_ (**A**, **B**) Anti-Aβ 4G8 immunostaining of whole fly brains expressing either Aβ_1–40_ (**A**) or Aβ_1–42_ (**B**) at age of 1 day. (**C**) Quantification of the immunostaining signals for Aβ_1–40_ (*n*=3) and Aβ_1–42_ (*n*=5). The mean intensity of anti-Aβ immunostaining signals were normalized to the mean intensity of GFP signal. Data represent means±S.D. (**D**) RT–qPCR detection of Aβ transcript levels of Aβ_1–40_ or Aβ_1–42_ transgene expression in fly heads. Data represent means±S.E.M. (**E**–**G**) Anti-Aβ 4G8 immunostaining shows the distribution of Aβ_1–40_ (**E**) and Aβ_1–42_ (**F** and **G**) in brains from animals at 16 days. The arrows indicate a focal staining of Aβ_1–40_ (**E**) in contrast with more extensive staining of Aβ_1–42_ distributed along with intracellular vesicular structures (blue arrows, **F**), plasma membrane of cell bodies (white arrows, **F**) and also in neurites (blue arrow, **G**). The arrowheads (**G**) point out the co-localization of GFP puncta and Aβ_1–42_ staining. *P* values are two-tailed and obtained by Student's two-tailed *t* test. Scale bars are 5 μm.

**Figure 2:**
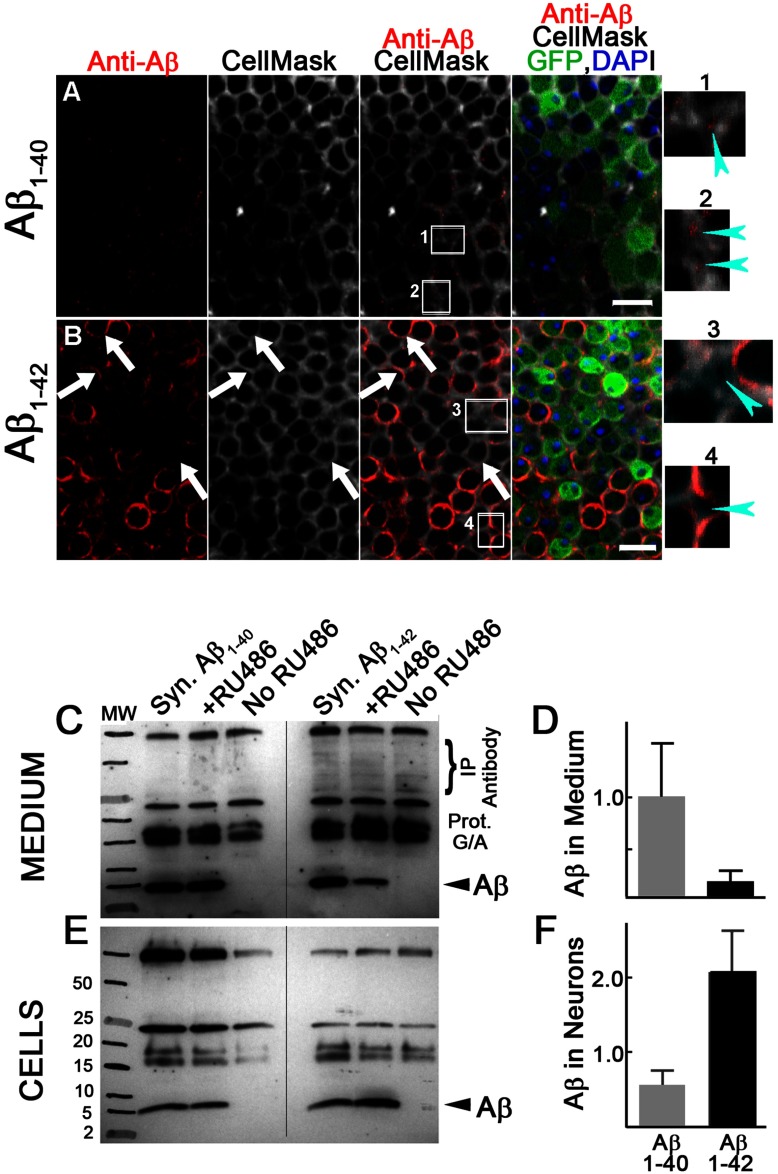
Aβ species-specific membrane association and secretion (**A**, **B**) Fly brains from 1-day-old animals were double-stained with anti-Aβ 4G8 antibody and CellMask, a plasma-membrane-binding fluorescent dye. Aβ_1–42_ staining (**B**) but not Aβ_1–40_ staining (**A**) co-localizes with the CellMask fluorescence. Arrows indicate the close association of Aβ_1–42_ staining with plasma membrane. The small panels on the right (1–4) are magnified views of the indicated regions shown in the CellMask column. Aβ_1–40_ staining (Panels 1–2) but not Aβ_1–42_ staining (Panels 3–4) appears in extracellular spaces where CellMask staining is absent (arrowheads). (**C**–**F**) Representative immunoprecipitation-Western blot images (**C**, **E**) and densitometric analyses (*n*=3 for **D**, *n*=4 for **F**) of Aβ_1–40_ and Aβ_1–42_ accumulating in culture medium (**C**, **D**) or within cells (**E**, **F**). Data represent means±S.D. *P* values are two-tailed and obtained by Student's *t* test. Scale bars are 5 μm.

Image analyses, including densitometric quantification of immunoprecipitation/Western blotting (Figures 2D and 2F), mean signal intensity of anti-Aβ immunostaining (Figure 1C), quantification of the mRFP-Rab4 objects (Figure 5D) and co-localization assay (Figure 5E), were performed using ImageJ (1.45s, NIH). Densitometric analysis of non-immunoprecipitation Western blotting was performed using Bio-Rad Quantity-One (Figure 5). The data analyzer was blinded to image identities relevant to experimental conditions. The sample sizes for biological replicates (*n*) are provided in relevant Figure legends. Data are presented as means±S.D. or S.E.M. as indicated. RT–qPCR data analysis for measurement of Aβ transcript levels (Figures 1D and 6D) was as described above. The normalized expression ratios are presented as means±S.E.M. For all statistical comparisons, two-tailed *P* values were obtained by Student's *t* test or ANOVA [corrected for multiple comparisons (Bonferoni) using GraphPad Prism5]. The α-level for all tests was set at 0.05.

## RESULTS

### Aβ_1–42_ appears to be selectively associated with membrane

Human Aβ_1–40_ or Aβ_1–42_, fused to a rat pre-proenkephalin secretory signal sequence (Finelli et al., 2004), were expressed separately in *Drosophila* neurons using the Gal4:UAS bipartite gene expression technique. Targeted neurons were additionally labeled by co-expression of soluble GFP which initially fills the cytosol and neurites and is detected by fluorescence microscopy (see Supplementary Figure S1). Anti-Aβ antibody 4G8 immunostaining was applied to whole brains to compare the initial accumulation and distribution of Aβ_1–40_ and Aβ_1–42_ peptide in brain region-matched samples from relatively young specimens (1-day-old, relative to eclosion, Figures 1A and 1B). The 4G8 antibody targets the 17th–24th amino acids common to both Aβ_1–40_ and Aβ_1–42_. The relative staining intensity appears significantly higher for Aβ_1–42_ than Aβ_1–40_ (Figure 1C). However, transcript levels of Aβ_1–42_ mRNA detected by RT–qPCR are lower than those of Aβ_1–40_ (Figure 1D). The distribution of Aβ_1–42_ staining is strikingly different compared with Aβ_1–40_ staining. Aβ_1–42_ staining appears to outline cell somas (Figure 1B). In brains taken from older specimens (16-day-old) Aβ_1–40_ staining appears to be mostly focal (Figure 1E) and remains less intense than Aβ_1–42_ (Figures 1F and 1G). In these older samples, Aβ_1–42_ staining still appears to be associated with some somal membranes, but staining is also evident in intracellular foci as well as in some likely neurites (Figures 1F and 1G). These intracellular foci were previously identified as dysfunctional autophagy–lysosomal vesicles and their number and size increases with age specifically in Aβ_1–42_-expressing samples (Ling et al., 2009). Our results suggest that Aβ_1–42_ may preferentially associate with various membranous structures, including plasma membranes, whereas Aβ_1–40_ does not.

### Neurons preferentially secrete Aβ_1–40_ but retain Aβ_1–42_

The C-terminus of Aβ_1–42_ has an additional isoleucine and alanine relative to Aβ_1–40_ that increases its hydrophobicity and membrane association (Marchesi, 2005). This increased membrane association may contribute to its greater accumulation within neurons by interfering with efficient secretion following biosynthesis through the secretory pathway. To obtain evidence for this possibility, we double-stained fly brains expressing Aβ with anti-Aβ 4G8 antibody and CellMask, a plasma membrane-specific fluorescent dye. In 1-day-old Aβ_1–42_ samples we observe extensive co-localization of antibody staining with CellMask staining (Figure 2B) suggesting an association of Aβ_1–42_ with plasma membranes. In contrast, Aβ_1–40_ samples exhibit only limited co-localization of a few plasma membrane foci along with some intracellular foci (Figure 2A). A close examination of Aβ_1–40_ staining reveals that additional Aβ_1–40_-positive signal is localized in regions not coincident with CellMask, but rather present in adjacent areas that may be extracellular spaces (Figure 2A, inserts 1 and 2). Aβ_1–42_ staining in contrast is not found in equivalent areas (Figure 2B, inserts 3 and 4) suggesting that Aβ_1–40_, but not Aβ_1–42_, may be more amenable to secretion into extracellular spaces. To test this possibility directly, we expressed Aβ_1–42_ or Aβ_1–40_ in primary cultured *Drosophila* neurons using a drug-inducible system to conditionally control expression (Supplementary Figures S1C and S1D). Temporal control of expression should minimize confounding factors that might result from specific neuronal toxicity of Aβ_1–42_. Quantification of Aβ in the culture medium or within neurons indicates that Aβ_1–40_ is preferentially recovered from culture medium while Aβ_1–42_ accumulates more prominently within neurons (Figures 2C–2F). These results support the proposal that neurons preferentially retain Aβ_1–42_, likely a consequence of its higher membrane affinity, but preferentially release Aβ_1–40_.

Movie 1

**Figure:** Movie 1: Morphological changes in cytosolic GFP redistribution in response to Aβ_1-40_ or Aβ_1-42_ expression GFP fluorescence was observed using confocal microscopy in brain region and age matched Aβ_1-40_ (the top panel) or Aβ_1-42_ (the bottom panel) samples. Image stacks representing an area of 47×30×15 µm (width×height×depth) in size were used to create the animations rotated from -90° to 90° around the Y axis. The Aβ_1-40_ sample has no apparent GFP puncta and shows relatively homogeneous GFP fluorescence. The Aβ_1-42_ sample exhibits the appearance of perinuclear punctuate GFP fluorescence representing dysfunctional AEL vesicles. The cell density and cytoplasmic GFP fluorescence in the Aβ_1-42_ sample is lower than the parallel Aβ_1-40_ sample because of widespread neurodegeneration (neuronal loss) and intraneuronal necrotic destruction. Fly age is 16 days.

### Vesicles accumulating punctate GFP and Aβ_1–42_ have endosomal properties

**Figure 3:**
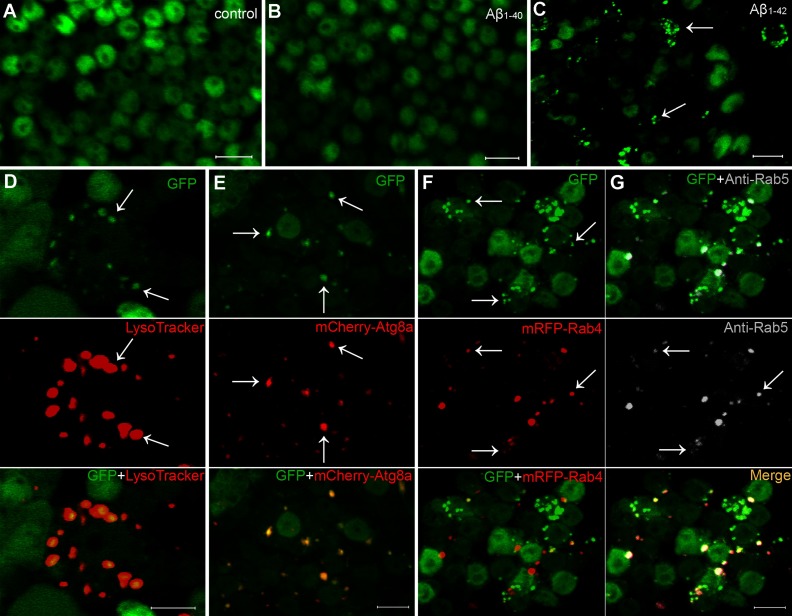
Aβ_1–42_ expression results in appearance of puncta with characteristic markers of the AEL pathway (**A**–**C**) Cytosolic GFP expressed in fly brains exhibits a homogenous distribution in control (**A**, no Aβ expression) or Aβ_1–40_ samples (**B**) but extensive punctate redistribution in Aβ_1–42_ samples (**C**). A 3D animated maximum intensity projection of the GFP fluorescence in the Aβ_1–40_ and Aβ_1–42_ samples shown in (**B**) and (**C**) is included as Supplementary Movie S1 (at http://www.asnneuro.org/an/006/an006e139add.htm). (**D**–**G**) Aβ_1–42_-induced GFP fluorescent puncta (arrows) co-localize with LysoTracker red staining (**D**), transgenic mCherry-Atg8a fluorescence (**E**), transgenic mRFP-Rab4 expression (**F**) and immunostaining of endogenous Rab5 (**G**). Note that the punctate distribution of mRFP-Rab4 co-localizes well with the positive anti-Rab5 immunostaining. Fly age is 16 days, expression controlled by Gad1-Gal4 driver. Scale bars are 5 μm.

A prominent morphological change induced by Aβ_1–42_, but not Aβ_1–40_, in our *Drosophila* model is a redistribution of the normal homogeneous cytosolic GFP fluorescence into punctate structures (Figures 3A–3C and Supplementary Movie S1 at http://www.asnneuro.org/an/006/an006e139add.htm). Our previous work established that these puncta are derived from an age-dependent autophagic sequestration of cytosolic GFP which is resistant to degradation by lysosomal hydrolases and is thus an indicator of long-lived dysfunctional autophagy–lysosomal vesicles (Ling et al., 2009). In many targeted neurons the contents of these vesicles leaks out into cytoplasm where it appears to initiate intraneuronal necrosis and a decrease in the fluorescence intensity of non-vesicular cytosolic GFP, possibly by reactivation of lysosomal hydrolases. This process increases the contrast of non-degraded GFP fluorescence contained within the vesicles relative to surrounding cytosolic GFP fluorescence (Ling et al., 2009; Ling and Salvaterra, 2011b).

Since Aβ_1–42_ is initially associated with plasma membrane and the autophagy and endosomal pathways converge (Eskelinen, 2005), we examined the possible contribution of endosomal involvement in the vesicle compartment recognized by punctuate GFP fluorescence and Aβ_1–42_ staining (Figure 1G). We first confirmed that GFP puncta substantially co-localize with LysoTracker red staining (a lysosomal marker indicating acidic pH, Figure 3D) and transgenic mCherry-labeled autophagy-specific gene 8a protein (mCherry-Atg8a, Figure 3E) identifying the contributions of autophagy and lysosomal vesicle fusion. In addition we observe that most of the GFP puncta also co-localize with transgenic mRFP-labeled Rab4 expression (Figure 3F) as well as anti-Rab5-specific antibody immunostaining (Figure 3G). Rab4 and Rab5 are endosomal markers (Sönnichsen et al., 2000) and these data thus identify the Aβ_1–42_-induced GFP puncta as dysfunctional AEL vesicles. Expression of cytosolic GFP alone or in combination with Aβ_1–40_, in contrast with the Aβ_1–42_ expression, does not result in accumulation of significant numbers of GFP puncta (Figures 3A and 3B and Supplementary Movie S1). Our results thus suggest that Aβ_1–42_, initially associated with plasma membrane, may be transported in part to AEL vesicles through the convergence of endosomal, autophagy and lysosomal structures. The absence of GFP catabolism, evident from its continued fluorescence in the AEL vesicles, along with decreased or absent turnover of the vesicles, appears to be a specific consequence of neuronal Aβ_1–42_ expression, but the specific catabolic inhibitory mechanism remains unknown.

### Inefficient vesicle turnover promotes continued fusion of AEL vesicles

**Figure 4:**
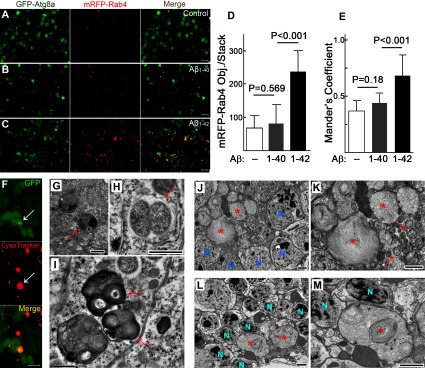
Aβ_1–42_ expression results in significant fusion of autophagy, lysosomal and endosomal vesicles (**A**–**C**) Representative images showing co-localization between mRFP-Rab4 labeled endosomal vesicles and GFP-Atg8a labeled autophagic vesicles in target neurons in brain region- and age-matched control (**A**), Aβ_1–40_ (**B**) and Aβ_1–42_ samples (**C**). Each image represents a maximum intensity projection of a stack of five confocal optical sections with 2 μm of depth. (**D** and **E**) Quantification of the mRFP-Rab4 puncta (**D**) and co-localization assay for mRFP-Rab4 puncta and GFP-Atg8a puncta (**E**) in image stacks. Each data point is from the analysis of nine image stacks collected from three to five individual brains with ten confocal optical sections in each stack (total depth of 4.5 μm). Data are means±S.D. Two-tailed *P* values obtained by Student's *t* test adjusted for multiple comparisons. (**F**) An AEL vesicle (arrow) likely derived from the apparent fusion of multiple vesicles (distinguishable as individual GFP puncta) with enlarged and unified LysoTracker red staining. Electron micrographs show the fusion of AEL vesicles (**G**–**I**) (arrows) that demonstrates either multiple sources of their contents (**G**), or distinguishable individual small vesicles (**H**), or a clear outline of sub-vesicle structures (**I**). (**J**–**M**) Large AEL vesicles may have developed through a process of continuous or unlimited vesicle fusion. The large AEL vesicles (asterisks) are several times larger than nearby AEL vesicles (**K**, arrows) and occasionally can appear even larger than an adjacent unaffected neuronal cell body (**L** and **M**). (**K**, **M**) is a higher power view of the AEL vesicles in (**J**, **L**). N, nuclei. Fly age=16 days, expression controlled by Gad1-Gal4 driver. Scale bars are 5 μm (**A**–**C**, **F**), 1 μm (**J**, **L**), or 0.5 μm (**G**–**I**, **K**, **M**).

Abnormal accumulation of autophagic and endosomal vesicles relevant to AD neuropathology has been postulated to result from a blockage of vesicle fusion with lysosomes, resulting in a failure to acquire catabolic enzymes necessary for cargo degradation (Funk and Kuret, 2012). We co-expressed GFP-Atg8a and mRFP-Rab4 in control, Aβ_1–40_ or Aβ_1–42_ animals to test if the decreased vesicle turnover in Aβ_1–42_-expressing neurons affects the convergence of autophagy and endosomal markers. Compared with age- and brain region-matched control samples with no Aβ expression (Figure 4A) or Aβ_1–40_ samples (Figure 4B), we observe enhanced accumulation of autophagic and endosomal vesicles in Aβ_1–42_ samples (Figures 4C and 4D). Since vesicle turnover is efficient in healthy neurons, this increased accumulation of AEL vesicles in Aβ_1–42_ samples suggests their turnover is slower and that they maintain an extended duration in cytosol. Co-localization of autophagic and endosomal markers is significantly greater in brains from Aβ_1–42_ flies compared with either control or Aβ_1–40_ flies (Figure 4E), suggesting that the decreased vesicle turnover in Aβ_1–42_ samples may actually promote fusion between autophagic and endocytic vesicles. In addition, we frequently observe complex intermediate structures that may be a manifestation of different stages of fusion between the diverse types of AEL vesicles in brain tissue in Aβ_1–42_ samples (Figures 4F and 4I). Some of these appear to be extremely large AEL vesicles (Figures 4J and 4M) that may result from a combination of extended vesicle duration and multiple vesicle fusion among dysfunctional AEL vesicles. In contrast, large AEL vesicles are absent from age-matched control or Aβ_1–40_ samples (Supplementary Figure S2 at http://www.asnneuro.org/an/006/an006e139add.htm).

### Decreasing AEL function results in decreased Aβ_1–42_ accumulation

**Figure 5:**
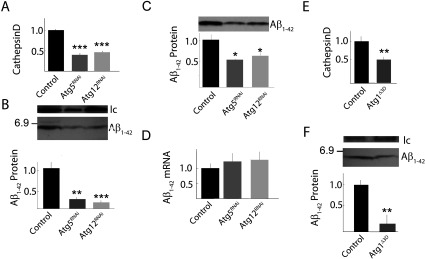
RNAi or loss-of-function genetic inhibition of autophagy results in decreased Aβ_1–42_ accumulation (**A**) Enzymatic activity of CathD is decreased in fly head extracts of samples expressing Atg5^RNAi^ or Atg12^RNAi^ relative to control samples with wild-type autophagy. Interfering RNA against *Atg5* or *Atg12* (RNAi) is targeted to the same neurons expressing Aβ_1–42_. *n*=5 for each data point. (**B**) Western blot image and densitometric analysis (*n*=5, bottom) of RIPA/SDS-soluble Aβ_1–42_ accumulation. The loading control (lc, actin) was used for densitometric normalization. The position of a 6.9 kDa molecular mass marker is indicated. (**C**) Western blot and densitometric analysis (*n*=3) of RIPA/SDS-insoluble, FA-soluble Aβ_1–42_ accumulation. (**D**) Aβ_1–42_ mRNA levels in fly heads measured by RT–qPCR. Data were normalized to expression levels of *Gapdh*. *n*=3. (**E**) CathD activity in fly heads with either wild-type autophagy (Control) or heterozygous for an *Atg1* loss-of-function allele (*Atg1^∆3D^*). *n*=3. (**F**) Western blot image (top) and densitometric analysis (*n*=3, bottom) of RIPA/SDS-soluble Aβ_1–42_ accumulation in brains from samples with wild-type autophagy (Control) or heterozygous for *Atg1* loss-of-function (*Atg1^∆3D^*). All data points are means±S.E.M. Statistical analysis by ANOVA for (**A**), (**B**) and (**C**) or Student's two-tailed *t* test for (**E**) and (**F**). *P<0.05,**P<0.01, ***P<0.001. Fly age=5 days. Expression controlled by Cha-Gal4 driver.

We decreased functional autophagy activity by expressing small interfering RNA transgenes (UAS-RNAi) targeting either autophagy-specific gene 5 (*Atg5^RNAi^*) or 12 (*Atg12^RNAi^*) in Aβ_1–42_-expressing neurons. Both RNAi genotypes exhibit a similar reduction in CathD activity (the major lysosomal aspartyl protease; Figure 5A) or the number of LysoTracker-positive structures (Supplementary Figure S3 at http://www.asnneuro.org/an/006/an006e139add.htm) suggesting successful reduction in both the function and catabolic capacity of convergent AEL vesicles. Expression of either RNAi transgene also results in decreased RIPA/SDS-soluble or FA-soluble Aβ_1–42_ accumulation (Figures 5B and 5C). Since the RNAi genotypes do not have significantly different levels of Aβ_1–42_ transcripts relative to control samples, the reduction in Aβ_1–42_ accumulation is likely to be post-transcriptional. The RNAi-mediated decrease in AEL function is limited to Aβ_1–42_-expressing neurons. We additionally used a conventional loss-of-function allele for the autophagy-specific kinase 1 gene (*Atg1^∆3D^*) that decreases AEL activity in all cell types (Scott et al., 2004). Heterozygous *Atg1^∆3D^* animals exhibit decreased CathD activity in fly heads as expected (Figure 5E) and also exhibit a reduction in accumulation of Aβ_1–42_ (Figure 5F). These data suggest the interesting possibility that AEL vesicle formation may be necessary for effective accumulation of Aβ_1–42_ in *Drosophila* neurons. It also seems likely that Aβ_1–42_ may in turn participate in development of the AEL dysfunctional vesicles.

### Aβ_1–42_ within AEL vesicles is recognized with aggregate-specific anti-Aβ antibodies

**Figure 6:**
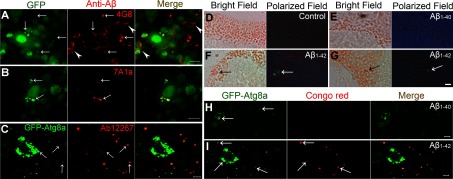
Selective deposition of amyloid Aβ_1–42_ in AEL vesicles (**A**–**C**) Brains from Aβ_1–42_ flies were immunostained using anti-Aβ antibody 4G8 (**A**), 7A1a (**B**) and Ab12267 (**C**). The arrows indicate co-localization between Aβ_1–42_ staining and GFP puncta (**A**, **B**) or GFP-Atg8a puncta (**C**). Note that the Aβ_1–42_ distribution detected by 4G8 staining is associated with ubiquitous intracellular membranes (**A**, arrowheads). In contrast, there is no additional staining beyond GFP puncta detected by the aggregate-specific 7A1a or the plaque-philic Ab12267 antibody. (**A**) is reused with permission from (Ling et al., 2009). (**D**–**G**) Brain sections from control (**D**), Aβ_1–40_ (**E**) or Aβ_1–42_ samples (**F**, **G**) are stained with Congo Red and viewed using polarized light. The control and Aβ_1–40_ samples show no congophilic staining. Congophilic staining is detected in Aβ_1–42_ samples (arrows, bright-field) that demonstrate typical apple-green birefringence (arrows, polarized field). Note that the congophilic staining also appears to have a punctate distribution (polarized field, **G**). (**H** and **I**) Brains from Aβ_1–40_ (**H**) and Aβ_1–42_ flies (**I**) were stained by Congo Red and observed using confocal microscopy according to (Wiesehan et al., 2003). The Congo Red fluorescence is observed in Aβ_1–42_ but not Aβ_1–40_ samples and co-localizes with the GFP-Atg8a puncta (arrows) in Aβ-targeted neurons. Fly age is 16 days (**A**–**C**, **H**, **I**) or 20 days (**D**–**G**). Scale bars are 5 μm.

Aβ_1–42_ is an aggregate-prone peptide and its formation into higher-ordered aggregation states is thought to be a key step in development of amyloid plaques in AD. To examine the relationship between dysfunctional AEL vesicles and Aβ_1–42_ amyloid-like aggregation, we performed immuno-staining of fly brains using anti-Aβ antibodies that recognize forms of Aβ. Immunostaining with anti-Aβ 4G8 antibody, which does not distinguish between diffuse and aggregated Aβ_1–42_, stains GFP puncta as well as plasma membranes and other types of fibrous-appearing intracellular Aβ_1–42_ structures (Figures 1G and 6A). However, immunostaining with aggregate-specific antibody 7A1a (Zhou et al., 2012) primarily co-localizes with GFP puncta (Figure 6B). A comparison of 4G8 and 7A1a immunostaining thus suggests that highly aggregated forms of Aβ_1–42_ may be limited to the dysfunctional AEL vesicles. This result was further confirmed by immunostaining with a different anti-Aβ_1–42_ antibody (AbCam, ab12267) with reportedly high affinity for plaque-like aggregations of Aβ_1-42_ (http://www.abcam.com/beta-amyloid-1-42-antibody-ab12267.html). The ab12267 immunostaining co-localizes well with many of the AEL vesicles identified by expression of transgenic GFP-Atg8a protein (Figure 6C). Our results thus suggest that aggregated forms of Aβ_1–42_ with potential amyloid-like properties are selectively associated with the dysfunctional AEL vesicles. The non-AEL-localized Aβ_1–42_ is in a non-aggregated state, a result that suggests the vesicles themselves may contribute to amyloid-like aggregation.

### AEL vesicles have typical amyloid plaque-like features

This focal aggregation of Aβ_1–42_ specifically within dysfunctional AEL vesicles could be a source of amyloid plaques. To test this, we performed Congo Red histochemical staining on brain sections. Congo Red staining that also exhibits apple-green birefringence is considered to be a ‘gold standard’ for identification of amyloid plaques in human tissue (Sipe et al., 2010). Brain tissue from Aβ_1–42_ flies, but not age-matched control (Figure 6D) or Aβ_1–40_ flies (Figure 6E), exhibits congophilic staining with typical apple-green birefringence when observed under polarized light microscopy (Figures 6F and 6G) suggesting an Aβ_1–42_-specific amyloid-type deposition. In addition, the congophilic staining appears to be discrete (Figure 6G) and thus consistent with localization restricted within AEL vesicles. To confirm the association between amyloid formation and AEL vesicles, we applied fluorescent Congo Red staining followed by confocal microscopy as described (Wiesehan et al., 2003). Congo Red fluorescence co-localizes in part with the punctate redistribution of transgenic GFP-Atg8a in Aβ_1–42_ but not Aβ_1–40_-targeted neurons (Figures 6H and 6I). These observations support the possibility that AEL vesicles containing aggregated Aβ_1–42_ deposits could potentially be a source of extracellular amyloid plaques.

### AEL vesicles may relocate from intra- to extra-cellular spaces

**Figure 7:**
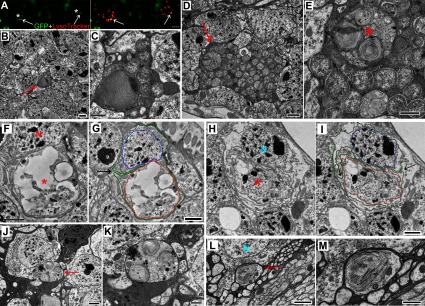
The AEL vesicles may relocate from intracellular to extracellular spaces (**A**) In older (16 day) flies, cytosolic GFP fluorescence in dying neurons (stars) becomes undetectable; however, brightly fluorescent dysfunctional AEL vesicles remain and they co-localize with LysoTracker staining (arrows). (**B**–**E**) Electron micrographs from Aβ_1–42_ flies showing clusters of AEL vesicles present in extracellular spaces (**B** and **D**, arrow; **C** and **E**, higher power view). Note that multiple AEL vesicles appear to be contained within a larger vesicle (**E**, asterisk). A multilamellar structure (**E**, asterisk) indicates a lipidic microenvironment inside the AEL vesicle. (**F** and **G**) A neuron appears to be budding off a large AEL vesicle (**F**, asterisk) through folding of the plasma membrane (**G**, arrow). (**H** and **I**) A large AEL vesicle separated (**H**, star) through plasma membrane infolding (**I**, the arrows). The colored dotted lines (**G**, **I**) trace the plasma membrane (green), the nuclear membrane (blue) and the AEL vesicle membrane (red). (**J**–**M**) Individual large AEL vesicles (**J** and **L**, the arrows) are localized in extracellular spaces. (**K**, **M**) A higher power view of the AEL vesicles. Note that neurons adjacent to these AEL vesicles have no apparent damage. N, nuclei. Fly age=16 days. Expression controlled by Gad1-Gal4 driver. Scale bars are 5 μm (**A**), 1 μm (**B**, **D**, **J**, **L**) and 0.5 μm (**C**, **E**–**I**, **K**, **M**).

How could amyloid-containing intracellular AEL vesicles relocate to extracellular spaces? Our previous work showed that compromised AEL vesicles participate in a necrotic-type neurodegeneration in *Drosophila* (Ling et al., 2009; Ling and Salvaterra, 2011b). Intriguingly, nearly all cytosolic GFP fluorescence in some dying neurons eventually disappears; however, some fluorescent dysfunctional AEL vesicles remain (Figure 7A). This implies that the vesicles containing aggregated amyloid-like forms of Aβ_1–42_ could remain in brain tissue even after the complete necrosis of their host neuron. Consistent with this possibility, we observe clusters of AEL vesicles in extracellular spaces (Figures 7B–7E) that conceivably could have been localized in a now completely degenerated neuron.

The delayed or absent turnover of dysfunctional AEL vesicles, in conjunction with cumulative fusion of additional autophagy and/or endosomal vesicles, also apparently results in the formation of extremely large vesicles which we routinely observe in Aβ_1–42_-expressing samples (Figures 7J–7M), but not in age-matched control or Aβ_1–40_ samples (data not shown). In some cases, these extremely large vesicles appear to be coincident with a process of tortuous infolding of the plasma membrane of their host neuron (Figures 7F and 7G). This infolding process could also conceivably lead to an eventual complete separation of the enlarged vesicles from their resident neuron (Figures 7H and 7I), even in the absence of any neurodegenerative changes in nearby neurons (Figures 7J–7M) and thus result in their appearance in extracellular spaces Similar morphological data is also apparent in very old *Drosophila* even in the absence of Aβ_1–42_ expression and may thus be a consequence of a normal aging process (Ling and Salvaterra, 2011b).

## DISCUSSION

Direct expression of Aβ_1–42_ in *Drosophila* neurons is a well-studied model exhibiting many phenotypes with potential relevance to AD (reviewed in Iijima-Ando and Iijima, 2010; Moloney et al., 2009) including decreased lifespan, neurological deficits, amyloid-like deposition in brain, compromise of memory processes and age-dependent neurodegeneration (Iijima et al., 2004; Ling et al., 2009). Interestingly, these phenotypes do not usually result from Aβ_1–40_ expression, suggesting underlying Aβ_1–42_-specific neurotoxic mechanisms. One of the most striking cellular differences we observe between Aβ_1–42_ and Aβ_1–40_ is the early age association of Aβ_1–42_ with membrane structures, especially plasma membrane, and this specific association may be a key factor in its specific proteotoxicity in *Drosophila*.

### Levels of Aβ_1–42_ versus Aβ_1–40_

The higher Aβ_1–42_ protein immunocytochemical staining we observe in young fly brains compared with Aβ_1–40_ (Figure 1C) is surprising in light of expression of similar transcript levels for these transgenes (Figure 1D). Although this could be a result of technical limitations of whole-brain staining, another possible explanation is that preferential membrane interaction of Aβ_1–42_ may render it resistant to degradation. This possibility has been observed in other systems (Knauer et al., 1992; Burdick et al., 1997; Ling et al., 2009). Our results are also broadly in accordance with AD as well as some mammalian AD models where neurons preferentially produce Aβ_1–40_ from APP proteolysis (Hartmann et al., 1997), but paradoxically accumulate higher levels of intraneuronal Aβ_1–42_ (Gouras et al., 2000, 2005; Laferla et al., 2007). Similar differential accumulation levels have also been previously reported in *Drosophila* photoreceptor neurons (Finelli et al., 2004). The *Drosophila* model thus potentially recapitulates a key feature of AD, the predominant intracellular accumulation of Aβ_1–42_ over Aβ_1–40_. Interestingly, this phenotype is not dependent on amyloidogenic proteolytic processing of APP.

### Recycling endocytosis may contribute to accumulation of Aβ_1–42_ in AEL vesicles

Our previous work emphasized a prominent role for dysfunctional autophagy–lysosomal vesicles in Aβ_1–42_ accumulation as well as age-dependent pathogenesis (Ling et al., 2009; Ling and Salvaterra, 2011b). Here we extend these observations by showing that the dysfunctional vesicles also express endosomal markers and thus represent convergent structures of autophagy, endosomal and lysosomal vesicle fusions.

A key question is how endosomal vesicles participate in the specific accumulation of Aβ_1–42_ in the AEL vesicles. We can rule out AEL-dependent processing of APP since the only proteolytic processing necessary for Aβ_1–42_ production in *Drosophila* is removal of the secretory signal peptide during trafficking through the secretory pathway. The complete removal of signal peptide from both Aβ_1–40_ and Aβ_1–42_ in *Drosophila* (Iijima et al., 2004) confirms its successful transit and processing through the secretory pathway. Only Aβ_1–42_, however, associates with plasma membrane, especially at early ages, and specifically accumulates within dysfunctional AEL vesicles suggesting that recycling endocytosis of plasma membrane associated Aβ_1–42_ followed by normal endosomal trafficking may at least in part contribute to its accumulation within AEL vesicles. Additional Aβ_1–42_ may also accumulate within AEL vesicles directly as a result of autophagy. This may occur by virtue Aβ_1–42_ association with non-endosomal membrane structures, including mitochondria or non-AEL-related vesicles that are targeted to autophagosomes as part of normal neuronal homoeostatic mechanisms. Non-membrane-associated intracellular Aβ_1–42_ aggregates could also conceivably be targeted directly to the autophagy pathway, but our antibody staining only detects highly aggregated forms of Aβ_1–42_ when they are already incorporated within AEL vesicles, especially in older samples. The reduction in Aβ_1–42_ accumulation we observe following genetic down-regulation of autophagy activity is thus likely to be a consequence of reduced convergence of autophagy and endosomal vesicles. Our genetic results further suggest that AEL vesicles may be necessary for optimal Aβ_1–42_ accumulation in *Drosophila*, a somewhat counterintuitive proposal given the catabolic nature of normal AEL function. We note, however, that the decrease in Aβ_1–42_ accumulation is entirely consistent with our previous genetic observations that autophagy reduction in *Drosophila* lengthens lifespan and reduces the rate of age-dependent neurological deficits while pharmacologically increasing autophagy has an opposite effect in Aβ_1–42_-expressing animals (Ling et al., 2009). Dysfunctional AEL processes may thus not only be necessary for optimal and preferential Aβ_1–42_ accumulation, but also may be necessary for subsequent neurodegenerative mechanism(s) in *Drosophila*.

The relative importance of autophagy, endosomal or lysosomal vesicle trafficking for Aβ_1–42_ accumulation is unknown; however, future genetic studies using endosomal and autophagy loss-of-function alleles in various combinations could potentially establish this. A recent study has also demonstrated that autophagy inhibition results in decreased Aβ accumulation in mammalian neurons (Nilsson et al., 2013), whereas other studies have found potentially conflicting results (Pickford et al., 2008; Boland et al., 2010). Many of these differences could be a result of various experimental details such as species differences in the type of APP being expressed, the particular types of AEL stimulation or inhibition used, confounding factors related to unspecified amyloidogenic APP processing (i.e. whether Aβ_1–42_ or Aβ_1–40_ predominates) or even the age and stage of neuronal compromise in the cells or neurons being studied. In AD, as well as the majority of mammalian AD models, Aβ must be generated by amyloidogenic proteolytic processing of APP, a process that reportedly occurs at both plasma membrane (Armstrong, 1998; Takahashi et al., 2002b; Marchesi, 2005) as well as intracellular AEL vesicles (Cataldo et al., 2004; Yu et al., 2005). It remains uncertain if either site predominates quantitatively (Haass et al., 2012), but both intracellular and extracellular Aβ are believed to be important for Aβ proteotoxicity as well as intracellular accumulation (Gouras et al., 2005; Mohamed and Posse de Chaves, 2011). Importantly, intracellular Aβ accumulation appears to precede extracellular Aβ deposition as well as plaque formation in some models (Gyure et al., 2001; Knobloch et al., 2007) and Aβ_1–42_ is the primary form of peptide accumulating within mammalian neurons (Laferla et al., 2007), results consistent with our observations in *Drosophila*.

### Dysfunctional AEL vesicles may be the source of plaque-like structures

Our previously data (Ling et al., 2009; Ling and Salvaterra, 2011d), as well as data presented here suggest a potential AEL-dependent mechanism for extracellular plaque-like formation in Aβ_1–42_-expressing *Drosophila*. First, APP amyloidogenic proteolysis is not necessary. Both recycling endocytosis as well as autophagy could provide the necessary routes for Aβ_1–42_ accumulation within AEL vesicle compartments and the relative selectivity of Aβ_1–42_ over Aβ_1–40_ could result from its greater membrane association. In young animals, the Aβ_1–42_-containing AEL vesicles are capable of cargo digestion and would thus turnover at an appreciable rate. Aged animals, however, have a decreased efficiency for cargo degradation as a consequence of normal aging processes (Cuervo et al., 2005). In Aβ_1–42_-expressing *Drosophila* neurons the dysfunctional AEL vesicles persist for long durations, continue to fuse with additional endocytic, autophagic and lysosomal vesicles and can become extremely large. Since Aβ_1–42_ is also present in non-AEL locations in a non-amyloid form in *Drosophila* neurons, *in vivo* accumulation of Aβ_1–42_ alone does not appear to be sufficient for amyloid formation. Amyloid formation may rather be dependent on the acidic and lipidic microenvironment of AEL vesicles, conditions shown to be favorable for formation of toxic aggregated forms of Aβ in mammalian neurons (Su and Chang, 2001). We have previously shown that some AEL vesicles leak their contents into cytoplasm (Ling et al., 2009). This process might conceivably create a non-AEL acidic and lipidic microenvironment in cytoplasm that could also convert non-amyloid Aβ_1–42_ into an amyloid-like form that is subsequently reincorporated into AEL vesicles. Finally, dysfunctional AEL vesicles containing amyloid-like Aβ_1–42_ would be relocated to extracellular spaces by two distinct mechanisms: neurodegeneration itself or separation of large dysfunctional AEL vesicles through a process of plasma membrane infolding.

The origin of extracellular senile plaques in AD is still a matter of debate. A prevalent view holds that they are formed by an autonomous condensation of extracellular Aβ peptides released from plasma membrane proteolysis of APP (Armstrong, 1998; Fiala, 2007). Alternative views emphasize an intracellular origin, including generation of Aβ directly by AEL vesicles (Glabe, 2001). Our proposed AEL-relocalization model in *Drosophila* may also have relevance for AD and mammalian AD models (Takahashi et al., 2002a; D’Andrea et al., 2003). The congophilic staining in *Drosophila* is discrete and consistent with amyloid restriction to a vesicular structure. Similar microdeposits have been observed in aged transgenic mouse brains expressing mutant APP (Takahashi et al., 2004; Stokin et al., 2005). The long duration and continuing fusion of membrane vesicles involved in AEL formation could easily result in production of not only large-sized amyloid plaques, but also a diversity of plaques sizes with spherical shapes, a common feature of AD and other AD models including our *Drosophila* AD model (Fiala, 2007; Ling et al., 2009; Ling and Salvaterra, 2011b). This proposed plaque model is also consistent with lysosomal materials, damaged organelles and other intracellular contents associated with AD plaques (Suzuki and Terry, 1967; Cataldo and Nixon, 1990; Fiala, 2007). It could obviate the difficulties in reconciling the different proportions of Aβ species found in diffuse, primitive and mature dense-core plaques (Fiala, 2007) believed to represent different stages of extracellular plaque maturation (Armstrong, 1998) with observations that senile plaques themselves contain primarily Aβ_1–42_. The model could even explain why Aβ is constitutively produced in human brain and peripheral tissues from fetal stages to old age (Wegiel et al., 2007), but amyloid deposition, as well as plaque formation, are generally absent in young people. It is also consistent with the absence of plaques when non-secretory Aβ_1–42_ is expressed directly in mammalian neuronal cytosol (Jucker et al., 1992; LaFerla et al., 1995) as well as studies showing that both internalization of extracellular Aβ as well as plasma membrane binding are necessary for toxicity in various types of cultured mammalian cells and neurons, and that endocytosis appears to play an essential role in this toxicity (Simakova and Arispe, 2007; Friedrich et al., 2010). Finally, our proposal agrees with the recent observations that autophagy may be necessary for plaque formation in a transgenic mouse model of AD that is dependent on amyloidogenic APP processing (Nilsson et al., 2013).

## Online data

Supplementary data

Supporting Movie
