# Supplementary material for: Accumulation of amyloid-like Aβ1–42 in AEL (autophagy–endosomal–lysosomal) vesicles: potential implications for plaque biogenesis
Source: ASN Neuro. 2014 Mar 12;6(2):e00139. doi: 10.1042/AN20130044 (PMC4379859; doi:10.1042/AN20130044)
Supplement: Supplementary data [file an006e139add.pdf]

ASN NEURO RESEARCH ARTICLE

ASN NEURO 6(2):art:e00139.doi:10.1042/AN20130044

OPEN ACCESS

# Accumulation of amyloid-like A$\beta_{1-42}$ in AEL (autophagy–endosomal–lysosomal) vesicles: potential implications for plaque biogenesis

**Daijun Ling*[1], Martha Magallanes* and Paul M. Salvaterra*†[1]**
*Department of Neuroscience, Beckman Research Institute of City of Hope, Duarte, CA 91010, U.S.A.
†Irell and Manella Graduate School of Biological Sciences, City of Hope, Duarte, CA 91010, U.S.A.

## SUPPLEMENTARY DATA

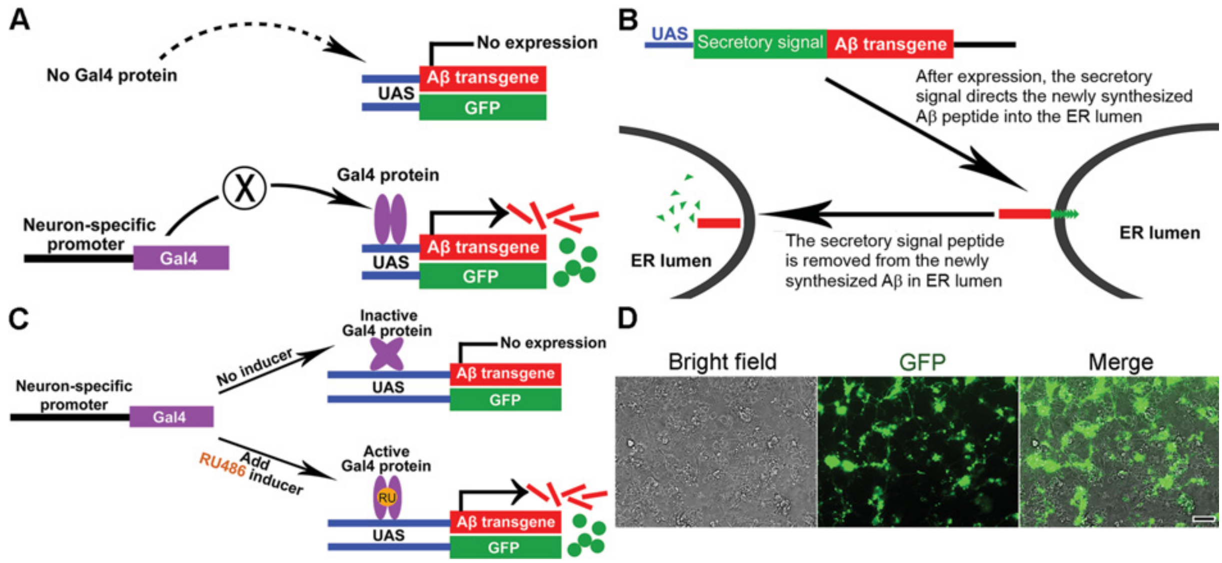


**Figure S1** ***In vivo* and *in vitro* expression of secretory human A$\beta_{1-40}$ or A$\beta_{1-42}$ in *Drosophila* neurons**
(**A**) Schematic illustration of neuron-specific expression of Aβ and GFP in *Drosophila* using the bipartite Gal4-UAS method. We used two different neuron-specific promoters fused to Gal4 in this study: a 3.1 kb Gad1-Gal4 transgene expressed only in GABAergic neurons or a 7.4 kb Cha-Gal4 transgene expressed only in cholinergic neurons. Either promoter drives expression in a large number of CNS neurons and both have been previously described (Ling et al., 2009). The driver stocks are genetically recombined with UAS-responder transgenes (i.e. UAS-Aβ containing a secretory signal sequence, UAS-GFP or UAS-fluorescently labeled fusion proteins). (**B**) Schematic illustration of the normal cellular processing of the Aβ secretory signal peptide. The peptide is completely removed when these constructs are expressed in *Drosophila* neurons (Iijima et al., 2004). Both A$\beta_{1-42}$ and A$\beta_{1-40}$ can then be freely secreted from the ER lumen. See a more detailed description in the Materials and Methods section of the main text. (**C**) Schematic illustration of mifepristone (RU486) inducible Gal4-UAS GeneSwitch method used to control temporal and neuron-specific expression of Aβ transgenes as previous described (Nicholson et al., 2008; Ling and Salvaterra, 2011b) and used in the cultured neuron experiments. (**D**) Representative images of primary cultured neurons. Expression of cytosolic GFP indicates an effective RU486 induction of transgenic neuronal Gal4 expression. Scale bar is 50 μm.

[1] Correspondence may be addressed to either of these authors (email psalv@coh.org or lingdaijun@gmail.com).

© 2014 The Author(s) This is an Open Access article distributed under the terms of the Creative Commons Attribution Licence (CC-BY) (http://creativecommons.org/licenses/by/3.0/) which permits unrestricted use, distribution and reproduction in any medium, provided the original work is properly cited.

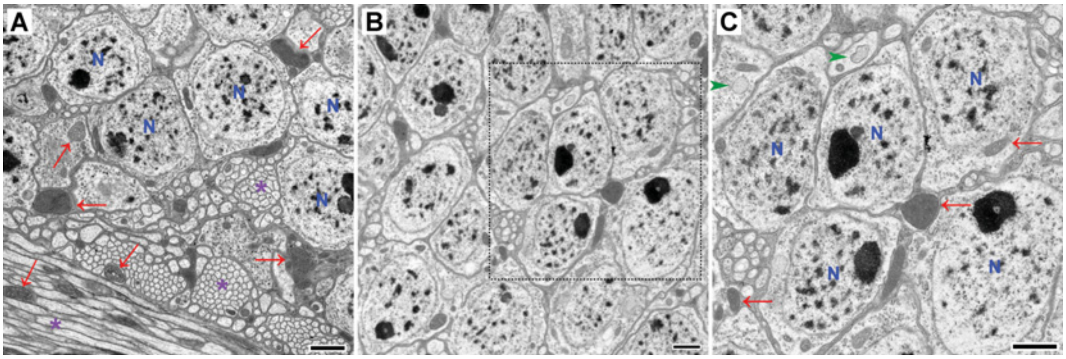


**Figure S2** **Representative electron micrographs of neurons from control samples (no Aβ expression) or $A\beta_{1-40}$-expressing samples**
(**A**) Control. (**B** and **C**) $A\beta_{1-40}$. (**C**) A higher magnification view of the square area indicated in (**B**). Note that the most prominent organelles in the cytoplasm of all three images appear to be mitochondria (arrows). No AEL vesicles are visible in control samples (**A**) and only a few small structures with the characteristic appearance of AEL vesicles are observed in intracellular spaces in (**C**, arrowheads). N, nucleus; asterisk, an axonal bundle. Fly age is 16 days. Scale bars are 1 μm.

© 2014 The Author(s) This is an Open Access article distributed under the terms of the Creative Commons Attribution Licence (CC-BY) (http://creativecommons.org/licenses/by/3.0/) which permits unrestricted use, distribution and reproduction in any medium, provided the original work is properly cited.

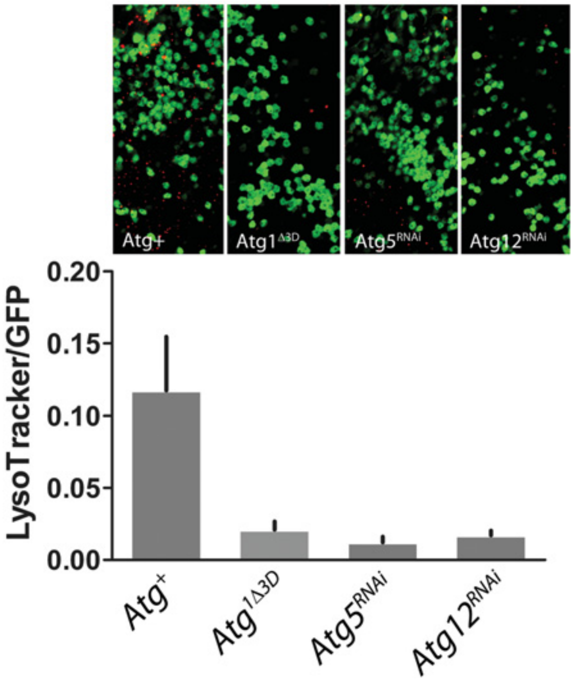


**Figure S3 Functional inhibition of autophagy decreases LysoTracker red staining**

Freshly dissected whole fly brains were incubated in PBS containing 0.5 μM LysoTracker red (Molecular Probes) for 5 min, washed twice with PBS and immediately observed using confocal microscopy (Zeiss LSM 510). Z-stacks of images were obtained using the green channel for GFP and the red channel for LysoTracker with a 63× (NA = 1.2) water-immersion objective. Image quantification for LysoTracker-positive objects was performed using ImageProPlus (Media Cybernetics). The experimenter was blinded to sample identities relevant to experimental conditions and independent observations were made on four individual brains for each experimental condition. The fluorescence area of LysoTracker-positive staining was normalized to the GFP fluorescence. Bars represent the mean normalized LysoTracker signal derived from analysis of at least ten optical sections from each brain. Error bars are S.E.M. ($n = 4$). The $Atg1^{\Delta 3D}$ heterozygotes as well as either RNAi genotype have decreased autophagy function and show less LysoTracker staining compared with the $A\beta_{1-42}$-expressing control sample with normal autophagy function ($Atg^{+}$). $P < 0.01$, ANOVA, Bonferoni correction for multiple comparisons. Fly age = 10 days, expression controlled by Gad1-Gal4 driver. LysoTracker staining apparently not associated with obvious GFP fluorescence is due to a combination of staining in non-targeted cells as well as weak or absent GFP fluorescence in cytoplasm of degenerating targeted neurons.

## REFERENCES

Iijima K, Liu H-PP, Chiang A-SS, Hearn SA, Konsolaki M, Zhong Y (2004) Dissecting the pathological effects of human Abeta40 and Abeta42 in *Drosophila*: a potential model for Alzheimer's disease. Proc Natl Acad Sci USA 101:6623–6628.

Ling D, Song H-J, Garza D, Neufeld TP, Salvaterra PM (2009) Abeta42-induced neurodegeneration via an age-dependent autophagic-lysosomal injury in *Drosophila*. PLoS One 4:e4201.

Ling D, Salvaterra PM (2011b) Brain aging and Aβ1–42 neurotoxicity converge via deterioration in autophagy-lysosomal system: a conditional *Drosophila* model linking Alzheimer's neurodegeneration with aging. Acta Neuropathol 121:183–191.

Nicholson L, Singh GK, Osterwalder T, Roman GW, Davis RL, Keshishian H (2008) Spatial and temporal control of gene expression in *Drosophila* using the inducible GeneSwitch GAL4 system. I. Screen for larval nervous system drivers. Genetics 178:215–234.

© 2014 The Author(s) This is an Open Access article distributed under the terms of the Creative Commons Attribution Licence (CC-BY) (http://creativecommons.org/licenses/by/3.0/) which permits unrestricted use, distribution and reproduction in any medium, provided the original work is properly cited.
